# Willingness to use smart fetal heart rate monitoring devices among pregnant women: an extension of the technology acceptance model

**DOI:** 10.3389/fpsyg.2024.1400720

**Published:** 2024-07-12

**Authors:** Shan Wu, Bingsheng Cui, Xiaofan Yu

**Affiliations:** ^1^School of Art, Jingchu University of Technology, Jingmen, China; ^2^Department of Industrial Design, Pukyong National University, Busan, Republic of Korea; ^3^Center for Postdoctoral Programme, Central Academy of Fine Arts, Beijing, China

**Keywords:** smart fetal heart-rate monitoring device, pregnant women, technology acceptance model, intention to use, structural equation model

## Abstract

The purpose of this study was to assess the significant factors that impact pregnant women’s willingness to use smart fetal heart-rate monitoring devices. We propose a research model that integrates technological factors (perceived compatibility and perceived credibility) and personal factors (health anxiety, personal physiological conditions, health consciousness, and health beliefs). The subjects of this study were Chinese women who were pregnant or had previously given birth. Data were collected and analyzed from 397 paper-and-pencil and electronic questionnaires. Our structural equation model indicated that perceived usefulness (β = 0.490, *t* = 7.591, *p* < 0.001), perceived ease of use (β = 0.352, *t* = 5.631, *p* < 0.001), health anxiety (β = 0.095, *t* = 2.664, *p* = 0.008), personal physiological conditions (β = 0.075, *t* = 2.142, *p* = 0.032), and health consciousness (β = 0.078, *t* = 2.110, *p* = 0.035) were the determinants of the intention to use smart fetal heart-rate monitoring devices, with perceived usefulness having the highest degree of influence. Furthermore, we discovered that the levels of perceived compatibility and perceived credibility did not have direct correlations with the intention to use these devices, but they did significantly influence the model. Perceived compatibility (β = 0.345, *t* = 6.601, *p* < 0.001) and perceived credibility (β = 0.519, *t* = 9.958, *p* < 0.001) significantly influences perceived ease of use. Perceived credibility (β = 0.421, *t* = 7.802, *p* < 0.001) significantly influences perceived usefulness. Based on these results, suggestions for future research are put forward.

## 1 Introduction

In 2022, China’s National Health Commission (NHC) stated in a press conference that China’s maternal mortality rate fell to 15.7 per 100,000, infant mortality rate to 4.9 per 1,000 (NHC, 2022); and in 2023, China’s National Bureau of Statistics (NBS) reported that the country’s national neonatal mortality rate was 3.1 per 1,000 (NBS, 2023). It follows that prenatal check-ups are essential to improve the quality and survival rate of newborns. The main monitoring indicator of fetal health is the fetal heart rate, which visualizes the functioning of the heart of the fetus through numerical values (Tan, 2014). When there is a significant change in the fetal heart rate, the doctor will consider not only possible heart diseases and dangers of the fetus but also the health status of the pregnant woman herself and give diagnostic recommendations (Tan, 2014). The implementation of fetal heart monitoring during pregnancy in women is of great practical importance to improve the quality of the fetus in the perinatal period and reduce neonatal mortality (Wu et al., 2017; Diotaiuti et al., 2022).

Currently, one of the main methods of non-invasive fetal heartbeat monitoring is fetal heart sound monitoring, which uses a fetal heart sound monitor to capture the frequency of ultrasound signals reflected from the fetal heartbeat and calculate the fetal heart rate based on the Doppler effect (Liu, 2012). Before monitoring the fetal heart, it is necessary to apply a coupling agent to the mother’s abdomen and keep moving the probe until the optimal monitoring site is found (Wu et al., 2017). The common home fetal heartbeat monitoring devices available in the market today are generally ultrasound-based. Therefore, it is necessary for pregnant women to use fetal heartbeat monitoring devices to protect their health and that of the fetus.

Advances in artificial intelligence technology have enabled smart wearable medical devices to surpass the boundaries of conventional medical devices and promote disease prevention and control. Tech companies are also placing more emphasis on the development and marketing of smart wearable medical devices that are valuable for in-home health monitoring and disease prevention (Yang et al., 2022b; Chen et al., 2023). Previous research on wearable medical devices has focused on the willingness to use them in the elderly population (Hsiao and Tang, 2015; Li et al., 2019; Mascret et al., 2020; Jeng et al., 2022; Chen et al., 2023). However, there has been little research examining whether pregnant women are willing to use smart wearable medical devices. Even fewer studies have focused specifically on pregnant women’s willingness to use smart fetal heart rate monitoring (SFHRM) devices. To fill this research gap, it is important to investigate the critical variables that may lead to increased adoption of SFHRM devices among pregnant women.

**FIGURE 1:**
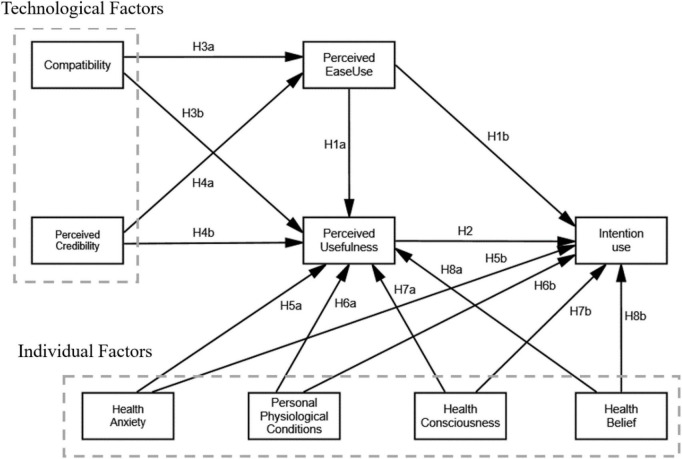
The research model.

SFHRM devices are defined as home medical devices that monitor the health of pregnant women and fetuses by adding artificial intelligence technology on top of fetal heartbeat sound monitoring devices, which can intelligently connect to smartphones to obtain detailed fetal heartbeat monitoring data, and also make them part of the internet of medical things for telemedicine and remote diagnosis (Yu, 2015), thus monitoring the health of pregnant women and fetuses. SFHRM devices are therefore very useful for pregnant women to monitor their health and the health of their fetus in real-time at home. To this end, we proposed a novel framework by integrating technology acceptance model (TAM) with technological factors (i.e., compatibility, perceived credibility), personal factors (i.e., health anxiety, personal physiological conditions, health consciousness, and health belief) (shown in Figure 1). The findings will promote the adoption of SFHRM devices among pregnant women, thereby effectively benefiting those with complex needs. Furthermore, product developers will be able to integrate these elements into the design of products to improve their effectiveness. Consequently, this research seeks to answer the following questions:

RQ1: How do technology-related factors affect the intention of pregnant women to use SFHRM devices?

RQ2: How do personal-related factors affect the intention of pregnant women to use SFHRM devices?

The remainder of this paper is structured as follows. The literature relating to relevant background questions is reviewed first, and then the model and hypotheses are presented. Subsequently, the approach taken to evaluate the hypotheses using the model is outlined. The findings of the examination of the structural equation model are then presented. Finally, the results of the analysis are discussed and conclusions are outlined.

## 2 Literature review and hypothesis development

### 2.1 The TAM

The TAM is frequently employed to elucidate users’ receptiveness and eagerness to employ information technology. Davis (1989) argued that the perceived ease of use and perceived usefulness of information technology have an effect on users’ attitudes, which in turn affects their intentions and behaviors. Perceived usefulness refers to the belief that the use of a technology will improve performance, while perceived ease of use refers to the belief that use of a technology is easy or requires little effort (Davis, 1989). The extent to which an individual will carry out a certain action in the future is known as their intention to use (Warshaw and Davis, 1985).

Due to its consistency, simplicity, and ease of extension, the TAM has been extended to the study of various high-tech products and services (Kim and Moon, 2023). Kim and Moon divided extended TAM studies into three categories: the first focuses on adding additional variables to the structure of the TAM framework; the second category adds predefined variables that affect perceived ease of use and perceived usefulness; and the last category adds moderating variables to the original structure (Kim and Moon, 2023).

The TAM is considered a precursor to technology-adoption modeling. Scholars have widely used the TAM framework to discuss the willingness to accept smart medical devices (Asadi et al., 2019; Chau et al., 2019; Wendland et al., 2019; Hayat et al., 2022; He et al., 2022; Rehman et al., 2022; Yang et al., 2022b; Attie and Meyer-Waarden, 2023; Kim, 2024). Rehman et al. (2022) employed the TAM framework to combine health characteristics (health beliefs and health information accuracy) with technology characteristics (compatibility and functional congruence). The results of their research demonstrated that the perceived ease of use and perceived usefulness of smart wearable healthcare devices have a direct influence on people’s willingness to use them. Kim and Moon (2023) investigated the effect of the occupations of female consumers on acceptance intentions for smart devices. Similarly, TAM has also been employed to investigate the perception of smartphone sleep apps before and after their use (Attie and Meyer-Waarden, 2023). Using the TAM as a base model, Asadi investigated consumer adoption of wearable medical devices in conjunction with other factors such as initial trust, compatibility, and health interest (Asadi et al., 2019). Jeng et al. (2022) considered the use of smart medical devices for e-health monitoring, finding that technology readiness and technological interaction had considerable impacts on perceived usefulness and ease of use. He et al. (2022) incorporated health benefits and hedonic motivation as moderating variables into the TAM framework to investigate consumers’ responses regarding the perceived ease of use and perceived usefulness of wearable devices for health monitoring during exercise by adding these innovative precursors. The TAM framework was enriched by their research, and their results confirm the impact of these factors on wearable health technology. Li et al. (2019) used the TAM framework to research health-monitoring systems, and they integrated external factors, health, and perceived risk into the TAM. Their findings demonstrated that perceived usefulness and perceived ease of use influence people’s inclination to use smart medical devices. Consequently, the following hypotheses are put forward.

H1a: Pregnant women’s perceived ease of use of SFHRM devices significantly affects their perceived usefulness.

H1b: Pregnant women’s perceived ease of use of SFHRM devices significantly influences their willingness to adopt them.

H2: Pregnant women’s perceived usefulness of SFHRM devices significantly influences their willingness to adopt them.

### 2.2 Compatibility

Compatibility (COM) refers to the level of compatibility of a new technology with a user’s needs, lifestyle, past experiences, and existing values (Payton et al., 2011). In the present study, compatibility refers to how well a pregnant woman’s work or life needs to match the functionality of an SFHRM device.

Li et al. (2019) monitored the health of older adults using wearable technology and found that compatibility significantly impacted perceived ease of use and perceived usefulness. Supposing consumers found that smart wearable healthcare devices are compatible with their smartphones or computers and that monitoring data can be transmitted to these devices, in this case, they may feel those devices compatible with their lifestyles and needs and will thus adopt it (Rehman et al., 2022). Compatibility is an important factor that affects a user’s perceived ease of use and the perceived usefulness of a product (Rehman et al., 2022). Other studies have found similar results with wearable medical devices (Hayat et al., 2022; Yang et al., 2022a). In previous studies, the user-perceived compatibility of a wearable healthcare product with a new technology is more likely to influence users’ willingness to use the product (Asadi et al., 2019; Wendland et al., 2019).

Smart medical systems also have to consider technical compatibility with smartphones, personal computers and wireless sensor networks, and whether health information can be transmitted to these devices (Li et al., 2019). Expectant mothers who perceive SFHRM devices as being suitable for their lifestyle, work approach, and requirements will be more inclined to use them. Consequently, we are of the opinion that compatibility will have a beneficial and noteworthy effect on perceived ease of use and perceived usefulness. Hence, the following hypotheses are proposed:

H3a: The compatibility of SFHRM devices positively affects their perceived ease of use by pregnant women.

H3b: The compatibility of SFHRM devices positively affects their perceived usefulness by pregnant women.

### 2.3 Perceived credibility

The trustworthiness of information technology in achieving its purpose is known as perceived credibility (PC) (Zhang et al., 2017; Chau et al., 2019). In recent decades, many studies have incorporated perceived credibility into various contexts such as on online banking (Luarn and Lin, 2005; Zhou et al., 2010) and mobile wallets (Shin, 2009). Previous research has integrated perceived credibility (which also increases when consumers have higher levels of innovation) into the TAM to investigate users’ willingness to adopt (He et al., 2022). Perceived credibility is judged by how secure and private a device is perceived to be by consumers, security is the safeguarding of data or systems from unapproved access or extraction, and privacy is the preservation of information gathered when a user interacts with a certain information system (Ariff et al., 2013).

When discussing wearable healthcare technology, it is significant to take into account both the perceived technical accuracy and the perceived privacy protection of these devices (Zhang et al., 2017). The former refers to the reliability of health-related data provided by smart wearable medical devices (Cheung et al., 2019). Wearable healthcare systems continuously detect and acquire users’ health data and analyze it to provide real-time health feedback, pathology diagnosis and other services. Consequently, the precision of the measurement of users’ health information is critical for gauging their health conditions (Chau et al., 2019). It is also essential to take into consideration the latter when using wearable medical technology, as users must enter comprehensive and detailed health information to gain access to more beneficial and tailored services. Thus, a user’s security concerns come from protecting personal information from unauthorized exfiltration (Zhang et al., 2017; Chau et al., 2019). The more credibility consumers attribute to wearable medical technology, the more useful they will find it. Research conducted by Zhang et al. (2017), Chau et al. (2019), He et al. (2022), and Rehman et al. (2022) demonstrated that perceived credibility has a beneficial effect on perceived usefulness. Based on these findings, we put forth the following hypotheses:

H4a: The perceived credibility of SFHRM devices has a beneficial impact on their perceived ease of use by pregnant women.

H4b: The perceived credibility of SFHRM devices has a beneficial impact on their perceived usefulness by pregnant women.

### 2.4 Health anxiety

Health anxiety (HA) is a condition in which individuals are extremely concerned about their health, and becomes extremely frightened when their bodies show symptoms that indicate illness, suspecting that they have a serious illness (Thatcher et al., 2007; Sang and Cheng, 2020; Chen et al., 2023). In the context of this research, HA refers to pregnant women’s worry or apprehension about their own health and that of their fetus during pregnancy. Pregnancy is a critical time in a woman’s life, and the physical and mental changes are more likely to produce anxiety, which in turn affect the development of the fetus (Diotaiuti et al., 2022). Individuals’ HA often promotes safety-seeking behaviors that are aimed at protecting and controlling their health and reducing health-related fears (Cabrera et al., 2020). HA has been shown to positively affect the desire to use wearable medical devices (Yang et al., 2022b; Chen et al., 2023).

Pregnant women with HA may obtain their body-related index parameters from SFHRM devices for self-assurance, as suggested by this paper. The more anxious pregnant women feel about their health, the more they find SFHRM devices useful, and the more likely they are to use them. Therefore, the following hypotheses are suggested:

H5a: The perceived usefulness of SFHRM devices by pregnant women is significantly enhanced by HA.

H5b: HA has a positive impact on pregnant women’s willingness to use SFHRM devices.

### 2.5 Personal physiological conditions

For older people, personal physiological conditions (PPC) refer to impairments in hearing, vision, language, cognition and memory that occur with age, as well as a range of difficulties they may have with the use of equipment (Chen et al., 2023). Significant changes in a woman’s physiological condition occur during pregnancy. Changes in hormone levels in the pregnant woman’s body can lead to systemic symptoms such as nausea and vomiting, fatigue, drowsiness, loss of appetite, and changes in food preferences. Changes can also occur in the body’s local systems such as tingling and enlargement of the breasts, frequent urination, and increased heart rate. The health status of both a pregnant woman and her fetus will change as a pregnancy progresses; therefore, SFHRM devices can help keep them healthy and safe. Previous research has shown that an individual’s health has a significant impact on their adoption of technology (Or et al., 2011; Chen and Chan, 2014). The PPC of elderly people have been found to have a considerable influence on their willingness to use wearable technology (Chen et al., 2023). However, Li et al. (2019) found that PPC can have a negative effect the perceived ease of use and perceived usefulness of SFHRM devices and people’s intentions to use them. The main reason for this paradoxical result lies the difference in age. Impaired health of people over 60 years of age may influence the acceptance of smart wearable systems to some extent (Li et al., 2019).

The present research concentrates on pregnant women or those who have undergone labor and delivery, and whose compromised health could potentially impact the acceptance of SFHRM devices. Therefore, the following hypotheses are formulated:

H6a: PPC negatively affect the perceived usefulness of SFHRM devices by pregnant women.

H6b: PPC positively affect pregnant women’s intentions to use SFHRM devices.

### 2.6 Health consciousness

Health consciousness (HCS) refers to individuals’ understandings of health, i.e., their habit of monitoring their state of health and taking appropriate actions based on that state of health (Michaelidou and Hassan, 2008; Chen and Lin, 2018; Srivastava et al., 2022). Health consciousness in relation to pregnant women means that pregnant women regularly monitor their own health and that of their fetus, and take actions to maintain their health. For example, when she finds that her fetus is squeezing around the neck, she maintains her health by observing the fetal movements through fetal heartbeat monitoring or seeking timely medical attention. HCS is necessary to develop the habit of self-examination of one’s health to make appropriate and timely efforts to maintain one’s state of health. Individuals who are mindful of their wellbeing will be more mindful of their health issues and take steps to safeguard their health (Dutta-Bergman, 2005; Pu et al., 2020). Such people are able to obtain health-related knowledge through a range of avenues, such as their social circles, the internet, etc., and they are more likely to become familiar with and use technologies and gadgets associated with maintaining good health (Dutta-Bergman, 2005). Increasing HCS will result in users having a more favorable outlook toward health-management tools (Zhu et al., 2023). Put simply, if users have faith in the efficacy of health-monitoring devices for addressing health concerns, they will use them and adopt more health-conscious measures. Most studies suggest that people who are conscious of their wellbeing are more likely to use health-surveillance tools to prevent any potential health problems (Srivastava et al., 2022; Yang et al., 2022b,c; Zhu et al., 2023). When a pregnant woman has a stronger HCS, her perceptions of SFHRM devices and intentions to use them increase. Consequently, this research puts forward the following hypotheses:

H7a: HCS has a positive impact on the inclination of pregnant women to use SFHRM devices.

H7b: The intention of pregnant women to use SFHRM devices is positively influenced by a heightened sense of HCS.

### 2.7 Health belief

Health belief (HB) is derived from the health belief model which posits that the health-related behaviors of individuals are influenced by four primary factors: susceptibility (people’s perceptions of having a particular disease), benefits (personal judgmentof the strengths of available alternatives that address perceived susceptibility and perceived severity), severity (individuals’ perceptions of the severity of health problems), and barriers (negative impacts of health actions considered by individuals) (Rehman et al., 2022). These factors refer to an individual’s perception of the effectiveness of a particular behavior in improving health (Zhang et al., 2017).

Previous research has investigated the link between a user’s HB and their willingness to use smart healthcare technology products by including HB in a TAM model, and the role of HB in reinforcing consumer intentions to use smart healthcare technology products has been validated (Zhang et al., 2017; Chau et al., 2019; He et al., 2022; Rehman et al., 2022). SFHRM devices can intelligently connect to a smartphone to obtain detailed fetal-heartbeat data. They are suitable for monitoring a baby’s heart rate during a whole pregnancy, preventing intrauterine distress and hypoxia, and making it easy for doctors to judge a baby’s health status, thus reducing health costs and increasing health benefits. Additionally, precious fetal sounds can be saved and shared with family members at any time. Given the potential health benefits of using such devices, pregnant women are likely to increase their willingness to adopt their use. Taking into account the available data, the following hypotheses were formulated:

H8a: The perceived usefulness of SFHRM devices by pregnant women is greatly influenced by HB.

H8b: The intention of pregnant women to use SFHRM devices is greatly influenced by HB.

## 3 Methodology

### 3.1 Participants

In line with the requirements of the research, women who were pregnant or had experienced childbirth were selected as respondents for this study. All participants volunteered to take part in the study after being informed of the purpose of the study and they were asked if they were pregnant or had given birth. To ensure the accuracy of the study, online questionnaires were distributed to women attending obstetrics and gynecology departments in hospitals in four cities in China. A total of 410 questionnaires were sent. Thirteen of these were disregarded due to inadequate completion and convergent responses. Finally, 397 questionnaires that met the criteria were taken into account for the analysis. Sample size meets requirements (Chin, 1998; Christopher Westland, 2010). Descriptive statistics of the participants are listed in Table 1. These indicate that 82.10% were currently pregnant, 17.90% had given birth, 48.90% were under 35 years old, 44.10% were between 35 and 50 years old, and 7.10% were over 50 years old. An SFHRM device was used by 40.3% of the participants (Supplementary note: fetal heart monitors are also used when pregnant women undergo medical checkups in hospitals).

**TABLE 1:** Participants’ demographic information.

Item	Category	Number	Percentage
Age	18–34	194	48.9
	35–50	175	44.1
	>50	28	7.1
Employment	White collar	18	4.5
	Freelancer	66	16.6
	Housewife	28	7.1
	Government employee	32	8.1
	Professionals	130	32.7
	Others	123	31.0
Education level	High-school graduate	98	24.7
	University graduate	267	67.3
	More than graduate school	32	8.1
Monthly income	<3000	59	14.9
	2500–4999	175	44.1
	5000–9999	142	35.8
	>10000	21	5.3
Experience with SFHRM devices at home	Yes	160	40.3
	No	237	59.7

### 3.2 Instrument development

This research questionnaire was split into two sections. The first part sought to collect demographic statistics, including age, employment, education level, monthly income and experience with smart fetal heart monitors. The purpose of the second section was to gather information regarding the nine variables that could be assessed in this research. This section consisted of 27 items measuring agreement or disagreement using a five-point Likert scale that has been widely adopted by a large number of researchers (Maurer and Pierce, 1998), with 1 signifying “strongly disagree” and 5 signifying “strongly agree.” All items were sourced from prior research and were modified to suit the requirements of SFHRM devices (Davis, 1989; Gao et al., 2015; Tan and Ooi, 2018; Chau et al., 2019; Cheung et al., 2019; Alam et al., 2020; Papa et al., 2020; Park, 2020; Wang et al., 2020; He et al., 2022; Yang et al., 2022b; Chen et al., 2023). Two professional experts and two translators carefully translated all items into Chinese. After the translation, two researchers participated in the back-translation sessions to verify the translation results.

The research conducted by Davis (1989), Papa et al. (2020), and Park (2020) were used to measure the perceived ease of use and perceived usefulness of smart healthcare devices. Compatibility was measured using items from Tan and Ooi (2018), and compatibility positively influences perceived ease of use. Measurement items for perceived credibility were taken from the works of Chau et al. (2019) and He et al. (2022), which assessed the impact of perceived usefulness. The items measuring the intentions of pregnant women to adopt medical wearable devices were adapted from those used in previous studies (Gao et al., 2015; Alam et al., 2020; Wang et al., 2020). The items relating to HA, PPC, HCS, and HB were adopted from several previous studies (Cheung et al., 2019; Yang et al., 2022a; Chen et al., 2023), which examined the influences of these individual factors. Table 2 contains a comprehensive compilation of all the measurement items used in this study.

**TABLE 2:** Measurement items.

Construct		Questionnaire items	Source
Compatibility (COM)	COM1	I think using an SFHRM device suits my way of managing health at home.	Tan and Ooi, 2018
	COM2	SFHRM devices are very much compatible with my lifestyle.	
	COM3	Using an SFHRM device is compatible with all aspects of my current healthcare management.	
Perceived credibility (PC)	PC1	The data provided by the SFHRM device are in line with my personal health data.	Chau et al., 2019; He et al., 2022
	PC2	I would find the software system of the SFHRM device credible.	
	PC3	Adequate protection of my personal health information would make me more likely to use an SFHRM device.	
Perceived ease of use (PEU)	PEU1	Using an SFHRM device is easy for me.	Davis, 1989; Park, 2020
	PEU2	It is easy to let an SFHRM device to do the health management.	
	PEU3	Interacting with an SFHRM device does not require mental effort.	
Perceived usefulness (PU)	PU1	Using an SFHRM device would increase my productivity.	Davis, 1989; Papa et al., 2020
	PU2	Using an SFHRM device would enhance the effectiveness of my monitoring of the health of my unborn child.	
	PU3	I find an SFHRM device useful in health monitoring.	
Intention to use (IU)	IU1	I will always try to use an SFHRM device to manage my health and that of my unborn child during pregnancy in the future.	Gao et al., 2015; Alam et al., 2020; Wang et al., 2020
	IU2	I plan to recommend others to use an SFHRM device.	
	IU3	I predict I will purchase an SFHRM device to manage my health information and that of my unborn child.	
Health anxiety (HA)	HA1	I am usually anxious about my health and that of my unborn child.	Yang et al., 2022b; Chen et al., 2023
	HA2	I frequently worry about my health and that of my unborn child.	
	HA3	If I hear about a certain disease, I think that I have it myself.	
Personal physiological conditions (PPC)	PPC1	My physical condition makes my daily activities strenuous.	Chen et al., 2023
	PPC2	My physical condition limits the kinds of activities I can do.	
	PPC3	My physical condition makes my daily activities difficult.	
Health consciousness (HCS)	HCS1	I am actively engaged in the prevention of disease and illness.	Yang et al., 2022b
	HCS2	I think taking preventive measures helps me to stay healthy.	
	HCS3	Living a healthy life is important to me.	
Health belief (HB)	HB1	I realize that bad living habits will cause harm to my health.	Cheung et al., 2019
	HB2	I perceive that bad living habits will cause harm to my health.	
	HB3	I think I can improve my health status effectively in many ways, such as by engaging in sports.	

### 3.3 Data analysis

This study applied AMOS (version 24.0) and SPSS (version 20.0) for data analysis. The specific analysis process consisted of three steps. Firstly, the measurement model was tested by validated factor analysis. Then, a structural equation modeling analysis was carried out to test the research hypotheses. Thirdly, analyses of direct, indirect, and total effects between variables were conducted to compare whether technological or personal factors were more important in influencing individuals’ intention to use. Results

### 3.4 Measurement model

CFA was used to confirm the reliability and validity of the constructs (Diotaiuti et al., 2021, 2020). The adequacy of the measurement model was evaluated using AMOS 24.0 based on the following indices: the model fit, standardized factor loadings, convergent validity, and discriminant validity.

To begin with, we examined the indices of the unconstrained model proposed by Hair et al. (2010), and the findings (CMIN = 862.585, DF = 305, CMIN/DF = 2.828, *p* < 0.000, RMR = 0.112, GFI = 0.866, NFI = 0.938, IFI = 0.959, RFI = 0.929, GIF = 0.866, AGFI = 0.833, CFI = 0.959, RMSEA = 0.068) confirmed that the model fit was satisfactory according to the criteria of Bagozzi and Yi (1988).

Subsequently, we assessed the reliability and validity of the constructs. The standardized factor loadings of all items exceeded 0.667 and they were statistically significant, surpassing the threshold value of 0.5 suggested by Schumacker and Lomax (2004). The results showed that the Cronbach’s alpha values of all the required measures exceeded 0.8. Therefore, the conditions for the reliability requirement were verified.

Composite reliability (CR) and average variance extracted (AVE) were used to assess convergent validity. Fornell and Larcker (1981) reported that the CR values for all the constructs should be higher than 0.70, and the AVE values should be higher than 0.50. These two criteria (Table 3) are met by all eight constructs in the current study. Consequently, it can be deduced that all components of the measurement model possess sufficient convergent validity.

**TABLE 3:** Results of construct validity and reliability analysis.

Latent Variable	Measurement Variable	Unstd.	S.E.	*t*-value	*P*	std.	α	CR	AVE
Compatibility	COM1	1.000				0.929	0.969	0.969	0.913
	COM2	1.084	0.028	38.980	***	0.957			
	COM3	1.076	0.025	42.582	***	0.980			
Perceived credibility	PC1	1.000				0.965	0.972	0.973	0.923
	PC2	1.050	0.018	59.794	***	0.990			
	PC3	1.041	0.026	40.146	***	0.926			
Perceived ease of use	PEU1	1.000				0.965	0.980	0.980	0.943
	PEU2	1.020	0.017	59.868	***	0.986			
	PEU3	0.996	0.020	50.386	***	0.963			
Perceived usefulness	PU1	1.000				0.917	0.960	0.961	0.892
	PU2	0.987	0.026	37.721	***	0.972			
	PU3	0.936	0.027	34.589	***	0.944			
Intention to use	IU1	1.000				0.919	0.922	0.926	0.806
	IU2	0.980	0.035	28.292	***	0.924			
	IU3	1.031	0.042	24.262	***	0.848			
Health anxiety	HA1	1.000				0.943	0.936	0.938	0.834
	HA2	0.993	0.028	35.504	***	0.956			
	HA3	0.871	0.034	25.495	***	0.836			
Personal physiological conditions	PPC1	1.000			0.904		0.947	0.948	0.858
	PPC2	1.017	0.035	29.017	***	0.906			
	PPC3	1.089	0.033	33.457	***	0.968			
Health consciousness	HCS1	1.000				0.776	0.832	0.844	0.648
	HCS2	1.055	0.070	15.069	***	0.947			
	HCS3	0.676	0.050	13.643	***	0.667			
Health belief	HB1	1.000				0.826	0.911	0.913	0.777
	HB2	1.030	0.048	21.428	***	0.880			
	HB3	1.062	0.047	22.507	***	0.936			

CR, composite reliability; AVE, average variance extracted;

***Significant at *p* < 0.001.

Additionally, the Fornell and Larcker (1981) criterion was used to determine if the AVE estimates for all constructs were greater than the squared estimates of inter-conceptual correlations, thus confirming discriminant validity. The discriminant validity for measurement items is presented in Table 4.

**TABLE 4:** Discriminate validity of the research model.

Construct	AVE	1	2	3	4	5	6	7	8	9
1. Perceived credibility	0.923	0.961								
2 Personal physiological conditions	0.858	0.194	0.926							
3. Health consciousness	0.648	0.517	0.148	0.805						
4. Health anxiety	0.834	0.238	0.369	0.312	0.913					
5. Health belief	0.777	0.211	−0.084	0.370	0.172	0.881				
6. Perceived usefulness	0.892	0.835	0.146	0.546	0.270	0.289	0.944			
7. Perceived ease of use	0.943	0.797	0.142	0.558	0.208	0.256	0.842	0.971		
8. Compatibility	0.913	0.808	0.145	0.566	0.362	0.269	0.767	0.766	0.956	
9. Intention to use	0.806	0.764	0.228	0.557	0.331	0.259	0.819	0.793	0.761	0.898

The findings suggest that the square root of the AVE values for each construct are greater than the absolute values of the correlation coefficients between that construct and the other constructs. Therefore, it is inferred that all factors in the measurement model have adequate discriminant validity.

### 3.5 Structural model

**FIGURE 2:**
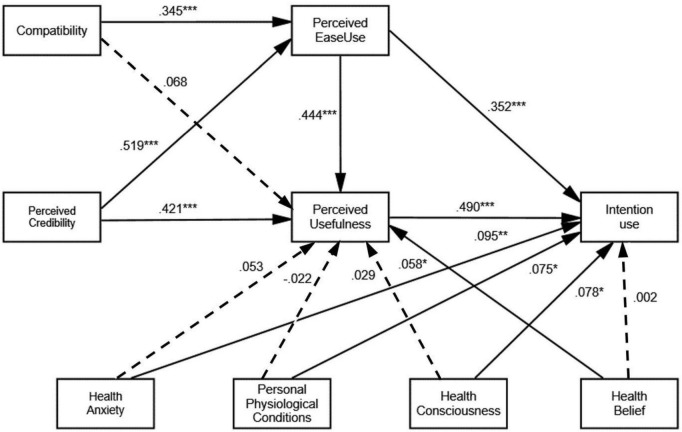
The research model with its standardized coefficients. ***p < 0.001, **p < 0.01, *p < 0.05.

The structural model was used to evaluate the connections between the constructs, both in terms of prediction and causality. The findings of an evaluation of the hypotheses using the structural model are displayed in Table 5 and Figure 2.

**TABLE 5:** Results of hypothesis testing.

Hypothesized path	Path coefficients	S.E.	*t*-value	*p*-value	Test result
H1a: PEU → PU	0.444	0.048	9.152	***	Supported
H1b: PEU → IU	0.352	0.057	5.631	***	Supported
H2: PU → IU	0.490	0.060	7.591	***	Supported
H3a: COM → PEU	0.345	0.055	6.601	***	Supported
H3b: COM → PU	0.068	0.053	1.339	0.181	Rejected
H4a: PC → PEU	0.519	0.051	9.958	***	Supported
H4b: PC → PU	0.421	0.052	7.802	***	Supported
H5a: HA → PU	0.053	0.025	1.714	0.087	Rejected
H5b: HA → IU	0.095	0.027	2.664	0.008**	Supported
H6a: PPC → PU	−0.022	0.016	−0.754	0.451	Rejected
H6b: PPC → IU	0.075	0.018	2.142	0.032*	Supported
H7a: HCS → PU	0.029	0.049	0.982	0.326	Rejected
H7b: HCS → IU	0.078	0.056	2.110	0.035*	Supported
H8a: HB → PU	0.058	0.031	1.961	0.050*	Supported
H8b: HB → IU	0.002	0.036	0.047	0.962	Rejected

PC, perceived credibility; PPC, personal physiological conditions; HCS, health consciousness; HA, health anxiety; HB, health belief; PU, perceived usefulness; PEU, perceived ease of use; COM, compatibility; IU, intention to use.

*Significant at *p* < 0.05,

** significant at *p* < 0.01,

*** significant at *p* < 0.001.

Drawing from our research, perceived ease of use significantly influences perceived usefulness (β = 0.444, *t* = 9.152, *p* < 0.001) and intention to use (β = 0.352, *t* = 5.631, *p* < 0.001), and thus H1a and H1b are accepted. Perceived usefulness was found to have a strong effect on intention to use (β = 0.490, *t* = 7.591, *p* < 0.001), supporting hypothesis H2. Perceived ease of use was found to be significantly impacted by compatibility (β = 0.345, *t* = 6.601, *p* < 0.001), while compatibility negatively affects perceived usefulness (H3b, β = 0.068, *t* = 1.339, *p* = 0.181); thus, H3a is supported and H3b is rejected. Perceived credibility significantly influences perceived ease of use (β = 0.519, *t* = 9.958, *p* < 0.001) and perceived usefulness (β = 0.421, *t* = 7.802, *p* < 0.001). Therefore, H4a and H4b are accepted.

Factors relating to the personal characteristics of pregnant women—HA (H5b, β = 0.095, *t* = 2.664, *p* = 0.008), PPC (H6b, β = 0.075, *t* = 2.142, *p* = 0.032), and HCS (H7b, β = 0.078, *t* = 2.110, *p* = 0.035)—positively affect the intention to use, while HB negatively affects the intention to use (H8b, β = 0.002, *t* = 0.047, *p* = 0.962).

In addition, HB (H8a, β = 0.058, *t* = 1.961, *p* = 0.050) also demonstrates a positive impact on perceived usefulness. But HA (H5a, β = 0.053, *t* = 1.714, *p* = 0.087), PPC (H6a, β = −0.022, *t* = −0.754, *p* = 0.451), and HCS (H7a, β = 0.029, *t* = 0.982, *p* = 0.326) negatively affect perceived usefulness.

### 3.6 Direct, indirect and total effects among the variables

After removing insignificant paths, direct, indirect and total effects were recalculated among the variables of compatibility, perceived credibility, personal physiological conditions, health consciousness, health anxiety, health belief, perceived usefulness, perceived ease of use, and intention to use (Table 6). Results showed that the total effect weights of technical factors (COM, PC, PEU and PU) on pregnant women’s willingness to use were 0.23, 0.502, 0.57 and 0.49 respectively, while the total effect weights of personal factors (HA, PPC, HCS and HB) on pregnant women’s willingness to use were 0.121, 0.065, 0.092 and 0.03 respectively. According to the results of the total effects, the four factors that had the greatest impact on intentions, in descending order, were PEU, PC, PU, and COM.

**TABLE 6:** Direct, indirect and total effects among the variables.

Dependent variable	Independent variable	Direct effect	Indirect effect	Total effect
PEU	PC	0.519	_	0.519
	COM	0.345	_	0.345
PU	PC	0.421	0.230	0.651
	PPC	−0.022	_	−0.022
	COM	0.068	0.153	0.221
	HCS	0.029	_	0.029
	HA	0.053	_	0.053
	HB	0.058	_	0.058
	PEU	0.444	_	0.444
IU	PC	_	0.502	0.502
	PPC	0.075	−0.011	0.065
	COM	_	0.230	0.230
	HCS	0.078	0.014	0.092
	HA	0.095	0.026	0.121
	HB	0.002	0.028	0.030
	PEU	0.352	0.218	0.570
	PU	0.490	_	0.490

PC, perceived credibility; PPC, personal physiological conditions; HCS, health consciousness; HA, health anxiety; HB, health belief; PU, perceived usefulness; PEU, perceived ease of use; COM, compatibility; IU, intention to use

## 4 Discussion

### 4.1 Impact of technology-related factors

This study proposes an extended integrated TAM model to explore the influencing factors affecting the use of SFHRM devices by pregnant women. The impact of technology-related factors is analyzed below.

The first observation from this analysis is that pregnant women’s perceived credibility of SFHRM devices significantly predicted perceived ease of use and perceived usefulness. This is consistent with existing findings (Li et al., 2019; Hayat et al., 2022; Rehman et al., 2022; Yang et al., 2022b). That is, the more pregnant women trust the SFHRM device, the more they find it useful and easy to use. Therefore, the perceived credibility of the SFHRM device by pregnant women is desirable and facilitates the adoption of the SFHRM device by pregnant women.

The second observation is that the greater the compatibility of SFHRM devices, the greater their perceived ease of use; i.e., the more that pregnant women found that the SFHRM devices fitted their needs, working style, and lifestyle, the easier they found them to use. However, perceived compatibility does not directly affect perceived usefulness, which is inconsistent with the previous hypothesis (Hayat et al., 2022; Rehman et al., 2022; Yang et al., 2022b). We now provide a possible explanation for this. Firstly, SFHRM devices are targeted at a specific population and there may be population variability. Its compatibility does not directly affect pregnant women’s perception of the device’s usefulness. And there may be indirect effects of other mediating factors that need to be revisited in greater depth in subsequent studies. Furthermore, when it became easy for pregnant women to use SFHRM devices to access health information, this strengthened their connection with the fetus and made them feel more confident about their own health and that of the fetus.

The third observation was that it was technology-related factors that had the greatest impact on pregnant women’s intention to use SFHRM devices. That is, the easier the SFHRM device were perceived by pregnant women, the more credible they were, the more useful they were, and the more compatible the devices were, the more likely pregnant women intend to use them. Of these, the easier to use had the highest total effect weight. Therefore, technically, the ease of use of SFHRM devices should be improved while satisfying credibility, usefulness, and compatibility. Impact of individual-related factors.

In terms of individual factors, pregnant women’s HA, PPC, and HCS are important influences on their willingness to adopt SFHRM devices. Among these, HA significantly affects the willingness to adopt SFHRM devices, and PPC and HCS present about the same influence. However, these factors do not directly influence perceived usefulness, which is inconsistent with previous hypotheses (Li et al., 2019; Yang et al., 2022b). Pregnant women experience changes in their personal physiology after pregnancy, and these changes may cause anxiety; furthermore, those with a high level of HCS will also be concerned about their health condition. All three of the factors will thus motivate pregnant women to use SFHRM devices to monitor their health.

It was found that HB does not directly influence willingness to adopt SFHRM devices, but it instead directly influences perceived usefulness, which is also inconsistent with previous hypotheses (He et al., 2022; Rehman et al., 2022). A possible explanation for this is that pregnant women find SFHRM devices useful for monitoring their health but not necessarily effective for improving their health; the intervention of a healthcare professional is still required in case of an emergency, so they would prefer to consult with a healthcare professional rather than relying on healthcare devices.

## 4.2 Implications for practice

The implications of this document are numerous in theory. First, SFHRM devices can be promoted to optimistic pregnant women through various marketing channels. Product demonstrations can be enhanced before purchase for pregnant women who are uncomfortable and insecure so that users can quickly adapt to the new product, and the system security of SFHRM devices should be strengthened. Pregnant women should be able to easily access SFHRM devices through smartphone software and be confident that their personal privacy is not compromised.

Second, the interfaces of SFHRM devices should be simple and easy to use so that users perceive it as simple to operate or use. This will reduce pregnant women’s technology anxiety and enhance their desire to employ SFHRM devices.

Third, in response to changes in the physiological conditions of individual pregnant women, SFHRM devices can include other monitoring functions in addition to fetal heart-rate monitoring, such as blood oxygen, blood pressure, blood glucose, etc. And, it is necessary to realize remote connection between SFHRM devices and physicians to provide high-quality professional guidance and assistance to pregnant women.

## 5 Conclusion

This study examined the structural relationships among technological factors (COM and PC), individual factors (HA, PPC, HCS and HB), and pregnant women’s intentions to use SFHRM devices, based on the TAM. the main objective of this study was to examine the technical and personal factors that impact pregnant women’s intentions to use SFHRM devices. This research confirmed that: (a) the perceived credibility of SFHRM devices strongly predicted their perceived ease of use and perceived usefulness; (b) the compatibility of SFHRM devices strongly predicted their perceived ease of use, but did not predict their perceived usefulness; (c) HA, PPC and HCS predicted the intention to use SFHRM devices but did not predict their perceived usefulness; (d) HB did not significantly affect the intention to use SFHRM devices.

This research is not without its drawbacks. First, due to the special characteristics of the pregnant women group and the sharp decline in fertility in China, the sample selection in this study was not limited to women who are currently pregnant but also included women who had already had children. Further research could be conducted to examine the similarities and differences between these groups by restricting the sample to women who are currently pregnant. Second, the present study did not include the age and occupation of the participants as moderating variables. Future studies could analyze differences in willingness to use SFHRM devices among pregnant women of different occupations and ages.

## Data availability statement

The raw data supporting the conclusions of this article will be made available by the authors, without undue reservation.

## Ethics statement

The studies involving humans were approved by the Ethics Committee of Jingchu University of Technology. The studies were conducted in accordance with the local legislation and institutional requirements. Written informed consent for participation in this study was provided by the participants’ legal guardians/next of kin.

## Author contributions

SW: Writing – original draft, Formal analysis, Writing – review and editing, Funding acquisition. BC: Methodology, Resources, Supervision, Writing – review and editing. XY: Investigation, Data curation, Visualization, Writing – review and editing.
